# Editorial: Developmental Modification Under Biotic Interactions in Plants

**DOI:** 10.3389/fpls.2020.619804

**Published:** 2020-11-23

**Authors:** Shinichiro Sawa, Masa H. Sato, Bruno Favery

**Affiliations:** ^1^Graduate School of Science and Technology, Kumamoto University, Kumamoto, Japan; ^2^Laboratory of Cellular Dynamics, Graduate School of Life and Environmental Sciences, Kyoto Prefectural University, Kyoto, Japan; ^3^INRAE, Université Côte d'Azur, CNRS, ISA, Sophia Antipolis, France

**Keywords:** biotic interaction, developmental modification, plant development, nematode, insects, parasitic plants, legume-rhizobia symbiosis

In many ecosystems, plants represent the center of biological interactions. As the Earth entered the Cenozoic era, gymnosperm, and fern populations declined while flowering angiosperms became the dominant plant species. Plant pollination and seed dispersal mechanisms then evolved and diversified as animals began to feed on plant nectars and fruits. It is believed that these interactions drove the evolutions and diversities of both plants and other organisms. Even though many plant interspecific interactions have been studied, the molecular mechanisms regulating these interactions have not been characterized extensively. In many cases, plants secret various metabolites into the environment to act as signaling molecules detected by other organisms. Conversely, interactions with other organisms may also cause modifications in plant development, such as the generation of novel cell types and organs (Favery et al., 2020).

The first step of biological interaction between plants and other organisms, particularly parasites and pathogens, likely involves chemotaxis. Molecules secreted by plants often can act as guidance cues. Many sugars, organic acids, phenolics, amines, and phytohormones secreted by plants were shown to possess attractive properties to various animals and microorganisms, and some of their cognate receptors have also been identified (Tsai et al., 2019; Oota et al., 2020; Tsai et al.). Attractants are thought to consist of unique or unusual compounds that may explain parasite/pathogen's host range. However, it appears that most plant parasites and pathogens are in fact attracted to common compounds such as plant metabolites and/or plant hormones. Therefore, it seems the identities of attractants alone is not sufficient to explain plant parasite/pathogen's host range. Instead, the specific interactions between the host-secreted attractants and the parasite receptors may help to better understand how these interactions drove the evolution of both plants and their interacting partners (Tsai et al.).

After parasites infiltrate their plant hosts, they may modify the host's signaling pathways to aid their own infection. Plant-parasitic nematodes (PPN), which include cyst nematodes and gall-inducing root-knot nematodes, are both scourges for agriculture and interesting case studies for such host manipulations. These nematodes spend the majority of their life cycles in plant roots, where they induce the formation of specific feeding structures inhabiting specialized feeding cells. These feeding cells known as giant cells and syncytia are multinucleate, hypertrophied, and hypermetabolic (Favery et al., 2020). Transcriptomic analysis have shown that the induction of these feeding cells involved an extensive reprogramming of gene expression. Regulators of gene expression, such as the small non-coding microRNAs, were shown to be essential for PPN feeding site formation. These microRNAs are likely to be master regulators expression re-programming during PPN feeding structure formation (Jaubert-Possamai et al.). PPN have been documented to inject molecules, named effectors, to induce the differentiation of feeding cells (Mejias et al.), and to suppress the host plant's immune response (Sato et al.). These effectors, associate with specific host proteins, enabling them to hijack important processes for cell morphogenesis and physiology and/or immunity. Some PPN-resistant plants have evolved intracellular nucleotide-binding domain leucine-rich repeat (NLR)-type receptors, which would recognize PPN effectors and induce NLR-triggered immunity (Sato et al.).

Aside from nematodes, insects are the other major multicellular animal taxon that interact with plants. The feeding of plant tissues from within plants (endophagy) is common among insects. Nutrition appears to be the main driver for the evolution of endophagy, however competition reduction, water conservation, and predation avoidance may have played significant roles (Tooker and Giron). Meanwhile, plant signaling pathways upon insect herbivory, a cascade of events including phosphorylation of a subset of transcription factors (e.g., ERF13) by calcium-dependent protein kinases (CRK2 and CRK3), have also recently been characterized in Arabidopsis (Miyamoto et al.). These phytohormone-responsive CRKs may thus play major roles in the coordination of plant defense responses during insect herbivory. On the other hand, many social aphids are known to form elaborate galls on aerial plant organs, where up to thousands of individuals may occupy for up to over a year. Insect-induced galls are unique organs that provide both shelter and nutrients to plant-parasitic insects. In addition, aphids were also found to utilize the gall vasculature to remove wastes from the colony (Kutsukake et al.), suggesting the utilities of insect galls are indeed more complex than expected. Transcriptomic analysis of Chinese sumac (*Rhus javanica*) horned galls induced by aphids (*Schlechtendalia chinensis*) revealed that meristem, flower, and fruit development master regulators as well as biotic and abiotic stress-responsive genes were highly upregulated in aphid galls (Hirano et al.).

Not all plant parasites are animals. Limitations in soil fertility have influenced the diversification of nutrient acquisition strategies in plants, driven certain species to parasitize on other plants (Zemunik et al., 2015). About 4,000 species of parasitic plants have been documented from all regions of the world, which consists of ~1% of all flowering plants (Nickrent, 2002). Many parasitic plants are known to have wide host ranges and can infect many economically important crop plants (Lanini and Kogan, 2005). In particular, members of the Orobanchaceae family such as *Striga, Orobanche*, and *Phelipanche* are important pest worldwide. Some parasitic plants, such as those from the genus *Cuscuta* (family Convolvulaceae), develop disc-like structures known as holdfasts in response to light and tactile cues, which are used to adhere to host plants (Shimizu and Aoki). Most parasitic plants then develop the root-like haustoria from the inner cortex, and invade the apoplastic space of the host's root. Once the haustorium reaches the host's vascular tissues, the parasite connects its own vasculature to the host's to acquire nutrients (Yoshida et al., 2016). Haustorium development is induced by host-derived signal molecules, collectively called haustorium-inducing factors, which include several cell wall-derived quinones and phenolics. In addition, the plant hormone cytokinins were also shown to trigger haustorium formation (Goyet et al.). Transcriptomic analyses using an *in vitro Cuscuta campestris* haustorium induction system revealed that genes involved in vascular stem cell development and proliferation were up-regulated in the haustoria in the absence of host, whereas genes required for xylem vessel cell differentiation were up-regulated only after the haustoria made contact with the host xylem. These results suggest host-derived signals and physical contact with the host vasculature are likely required to initiate haustoria xylem differentiation through transcriptional regulation (Kaga et al.).

Plant interspecific interactions are not limited to herbivory and parasitism. One of the best-characterized symbiotic plant interspecific interactions is formed between leguminous plants and nitrogen-fixing rhizobia bacteria. These interactions take place in root nodules, which are unique root organs formed specifically to house the rhizobia. The host plants receive organic nitrogen from the rhizobia, in exchange for providing the rhizobia with carbohydrates as nutrients and nodules as accommodation. This mutualistic interaction has evolved sophisticated signaling networks that regulate rhizobia recognition, colonization, differentiation, and nodule formation. Cysteine-rich peptides, reactive oxygen/nitrogen species and toxin–antitoxin modules have all been documented to contribute to the regulation of legume-rhizobia symbiosis (Syska et al.). Interestingly, these molecules are typically known to be involved in anti-microbial immune responses, yet here they have evolved to accommodate rhizobia colonization by escaping the host plant's innate immunity (Syska et al.). Furthermore, two plant growth-promoting rhizobacteria (PGPR) strains, *Bacillus* sp. (12D6) and *Enterobacter* sp. (16i) were shown to be capable of rapidly colonizing the rhizosphere (Jochum et al.). Interestingly, inoculation of these PGPR strains to wheat (*Triticum aestivum*) and maize (*Zea mays*) rhizospheres significantly delays the onset of drought symptoms, which are likely due to root system architecture modifications induced by the PGPR (Jochum et al.).

The diverse collection of articles in this Research Topic highlights the vibrant and rapidly-changing research in the field of plant developmental modification during biotic interactions (Figure 1). These new findings contribute to the understanding of how species interactions influence the evolution and diversity across ecosystems.

**Figure 1:**
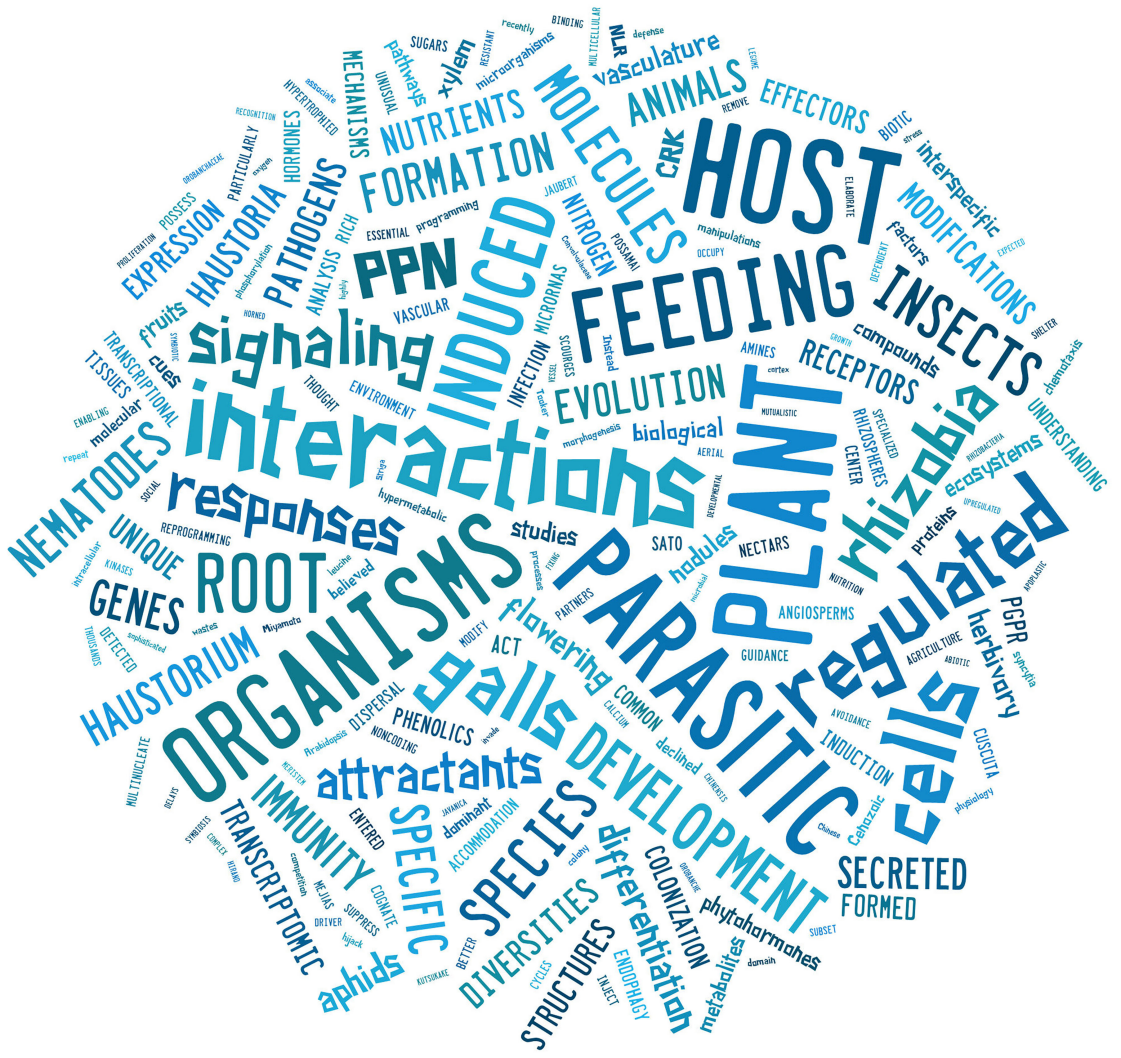
Word cloud summarizing this Research Topic on developmental modification under biotic interactions in plants. This visual display was made with tagxedo (http://www.tagxedo.com/) using the words of this editorial article.

## Author Contributions

All authors listed have made a substantial, direct and intellectual contribution to the work, and approved it for publication.

## Conflict of Interest

The authors declare that the research was conducted in the absence of any commercial or financial relationships that could be construed as a potential conflict of interest.
